# Antiviral cytotoxic T lymphocyte responses for long term prognosis of corneal infection by cytomegalovirus in immunocompetent subjects

**DOI:** 10.1038/s41598-022-09312-8

**Published:** 2022-03-30

**Authors:** Ryu Uotani, Dai Miyazaki, Yumiko Shimizu, Fumie Ohtani, Tomoko Haruki, Shin-ichi Sasaki, Ayumi Koyama, Yoshitsugu Inoue, Tatsuo Suzutani

**Affiliations:** 1 Division of Ophthalmology and Visual Science, Faculty of Medicine, Tottori University, 36-1 Nishi-cho, Yonago Tottori, 683-8504 Japan; 2 Fukushima Medical University, Fukushima, Japan

**Keywords:** Viral pathogenesis, Corneal diseases, Immunopathogenesis, Infection

## Abstract

Ocular cytomegalovirus (CMV) infections in immunocompetent individuals are rare, but its activation can cause chronic and relapsing inflammation in anterior segment of the eye resulting in loss of corneal clarity and glaucoma. Fifty five patients with anterior segment CMV infection were assessed for their clinical characteristics, and CMV corneal endotheliitis was found to cause significant loss of corneal endothelial cells. The disease duration with recurrences was significantly correlated with the maximum intraocular level of CMV DNA. To examine why CMV is activated in healthy immunocompetent individuals and causing corneal endothelial cell damage, assays of cytotoxic T cells (CTLs) which directly target infected corneal endothelial cells were performed for 9 HLA-matched CMV corneal endotheliitis patients (HLA-A*2402). When the cell loss was analyzed for associations with CTL responses, CMV-induced endothelial cell damage was mitigated by pp65-specific CTL induction. The recurrence-free time was also prolonged by pp65-specific CTL induction (hazard ratio (HR): 0.93, *P* = 0.01). In contrast, IE1-specific CTL was associated with endothelial cell damage and reduced the time for corneal transplantation (HR: 1.6, *P* = 0.003) and glaucoma surgery (HR: 1.5, *P* = 0.001). Collectively, induction of pp65-specific CTL was associated with improved visual prognosis. However, IE1-specific CTL without proper induction of pp65-specific CTL can cause pathological damage leading to the need of surgical interventions.

## Introduction

Cytomegalovirus (CMV) is a ubiquitous pathogen and more than 60% of the world population is seropositive for CMV^1^. In Asia and South America, almost 100% of the population is seropositive for CMV^1^. This infection is mostly silent although a higher mortality and health deterioration have been reported in elderly individuals with this infection^2–5^.

A representative manifestation of ocular CMV infection is retinitis which is observed in immunocompromised individuals, although CMV infections can also cause eye inflammation in immunocompetent individuals.

The eye is partitioned into the anterior and posterior segments by the lens-iris diaphragm, and CMV corneal endotheliitis affects mainly the anterior segment of the eye. The disease is chronic and recurrences occur throughout the lifetime of the affected individual. However, this disease entity is rare. In the 2004 to 2011 nationwide survey of the 1160 members of the Japan Cornea Society, only 106 patients with CMV endotheliitis were reported throughout Japan^6^.

Anterior segment diseases cause inflammation of the corneal endothelial cells, trabecular meshwork, and iris^7^. Endothelial inflammation or endotheliitis leads to a loss or dysfunction of the corneal endothelium. Because the corneal endothelial cells are an essential component for maintaining the clarity of the cornea, corneal endothelial cell damage leads to a clouding of the cornea which is diagnosed as bullous keratopathy. This is important because bullous keratopathy requires corneal transplantation for visual recovery.

The trabecular meshwork is the apparatus that controls the outflow of intraocular fluids. Its inflammation causes a dysfunction or a scarring of the outflow facility which results in a sustained elevation of the intraocular pressure (IOP). This increased IOP is diagnosed as secondary glaucoma which is an important cause of blindness^8^.

Topical or systemic antiviral drugs have been used to manage the CMV corneal endotheliitis in the anterior segment of the eye. However, these treatments do not eliminate CMV from the eye and reactivation still occurs. Thus, development of alternative preventive measures including vaccination are needed.

It also remains unclear why healthy subjects have a recurrence of this rare but visually devastating disease. Responses of anti-viral cytotoxic T cell (CTLs) responses are recognized as the most efficient and effective way to suppress CMV infection and its activation.

Of the viral antigens, pp65 has been recognized as a major target for the CD8 T and the CD4 cells for CMV infections^9^. Currently, studies on CTL responses and candidate viral proteins are not available for CMV infections of the eye.

Thus, the purpose of this study was to evaluate how the CTL response can target representative viral proteins in infected corneal endothelial cells, and to analyze how this response affects the outcome or prognosis of the CMV endotheliitis. We shall show that viral protein pp65-specific CTL responses reduce the number of recurrences and protect the cornea from the loss of endothelial cells. However, the IE1-specific CTL responses without proper induction of pp65-specific CTL were pathogenic and were associated with poor prognosis requiring corneal transplantation or glaucoma surgery. Our findings are consistent with the previous findings that pp65 can serve as a promising target of vaccination strategy for infectious diseases by CMV^9^.

## Results

### Characteristics of patients with anterior segment infection by CMV

**Figure 1:**
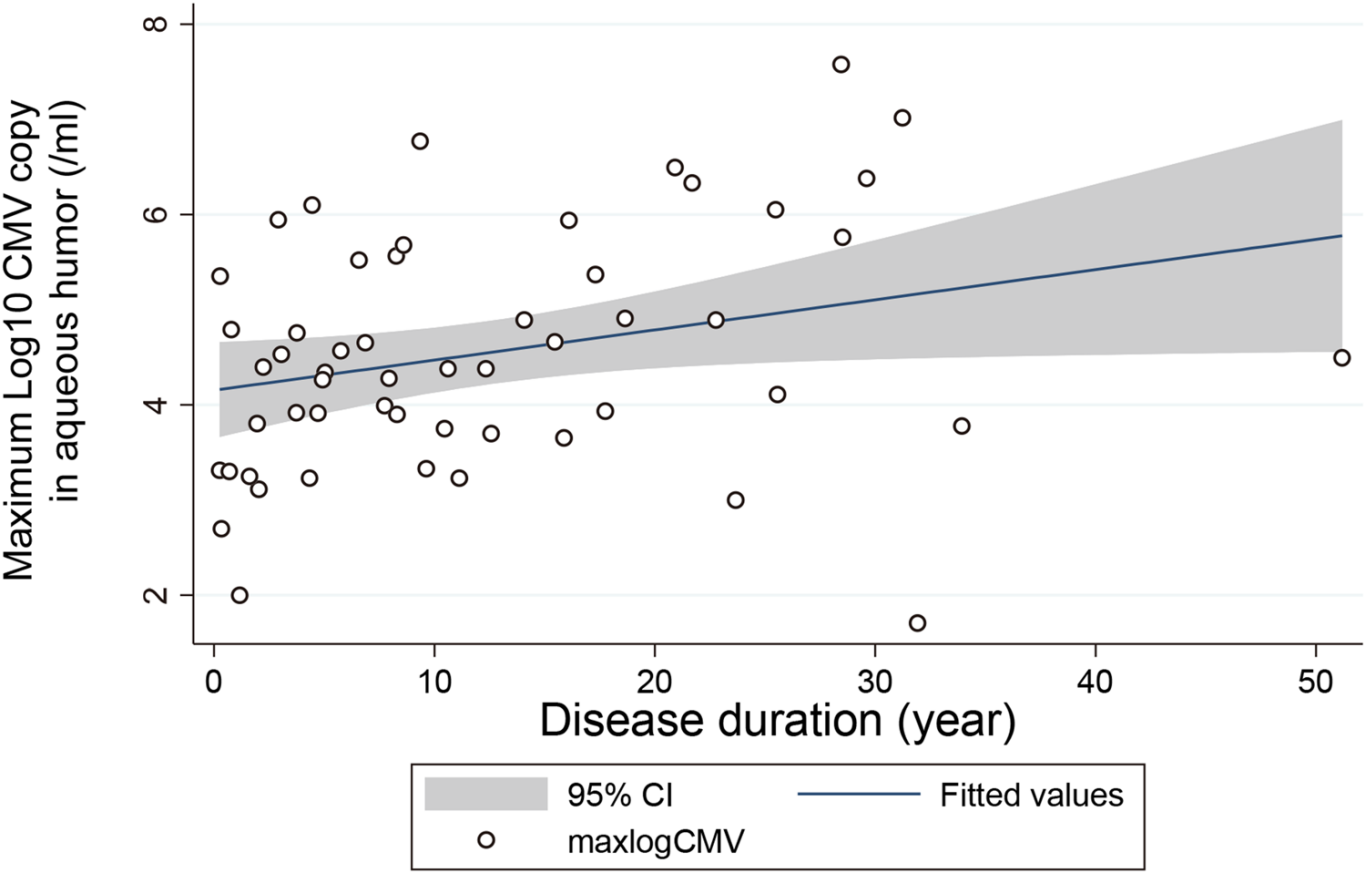
Association of disease duration and intraocular maximum copy numbers of the DNA of CMV in the anterior segment of CMV infected eyes. Fifty-five patients with CMV anterior segment infection were assessed for copy numbers of the DNAs of CMV in the aqueous humor. Maximum CMV copy numbers during the follow-up period was correlated with longer disease duration. Spearman correlation analysis, ρ = 0.32, *P* = 0.01.

Fifty-five patients with anterior segment infection by CMV were assessed. The mean age was 60.5 ± 14.5 years, and 11 patients (20.0%) were affected bilaterally (Table 1). The duration of the disease with recurrences was 12.5 ± 11.1 years, and the duration with recurrences was significantly correlated with maximum CMV copy numbers in the aqueous humor during the follow-up period (Spearman correlation analysis, ρ = 0.32, *P* = 0.01, Fig. 1).

**Table 1:** Patient Characteristics of corneal endotheliitis and anterior uveitis by cytomegalovirus infection.

	Corneal endotheliitis	Anterior uveitis	Total	*P* value
Number of patients	25	30	55	
Male	19	19	38	0.31
bilateral	6	5	11	0.5
Age (mean ± SD)	63.6 ± 11.1	58 ± 16.6	60.5 ± 14.5	0.15
Disease duration (year)	16.8 ± 12.3	8.8 ± 8.5	12.5 ± 11.1	0.006
Maximum CMV copy number in aqueous humor (/ml)	34,000 ± 15	35,000 ± 21	34,000 ± 18	0.99

Values were shown as mean ± standard deviation. Copy number was calculated after Log transformation.

Of the 55 cases of anterior segment CMV infections, 25 cases were corneal endotheliitis.

The mean duration with recurrences of the corneal endotheliitis was 16.8 ± 12.3 years, and it was significantly longer than that of anterior uveitis (8.8 ± 8.5 years; *P* = 0.006). The mean maximum copy number of the DNA of CMV in the aqueous humor (after log transformation) was 34,000 ± 15 copies/ml for the corneal endotheliitis patients and 35,000 ± 21 copies/ml for the anterior uveitis patients.

Because the corneal endotheliitis patients have a long duration of the disease with frequent recurrences and poorer visual outcomes^10^, we focused on the corneal endotheliitis.

### Induction of corneal endothelial cell-specific CTL responses in CMV endotheliitis patients

To determine how the infected immune system of the host protects the corneal endothelial cells from reactivated CMV, we determined the roles played by the anti-viral CTL responses targeting CMV-infected corneal endothelial cells.

To do this, we developed a CTL assay for corneal endothelial cells using HLA: A*2402^11^. Patients positive for A*2402 were selected for the CTL measurements. Of the 55 cases, 11 eyes of 9 patients were typed as A*2402 (Table 2). The mean maximum CMV copy number in the aqueous humor (after log transformation) was 180 000 ± 15 copies/ml in these patients. The mean age of the patients was 70.1 ± 8.5 years, and the duration of the disease was 20.9 ± 12.8 years. Recurrences were frequent, and the number of recurrence episodes was 5.9 ± 3.4 during the disease progression.

**Table 2:** Characteristics of CMV corneal endotheliitis patients measured for anti-viral cytotoxic T lymphocyte activity.

ID	Left/right	age	Gender	Disease duration (years)	Number of recurrences	Maximum CMV copy number in aqueous humor (/ml)
1	L	83	Male	18.6	5	8.1 × 10^4^
2	R	61	Male	14.1	5	7.8 × 10^4^
3	R/L	76	Male	12.3	5/4	3.0 × 10^2^/2.4 × 10^4^
4	R/L	65	Male	51.1	4/5	8.0 × 10^3^/2.5 × 10^5^
5	R	66	Male	20.9	4	3.1 × 10^6^
6	L	66	Male	17.8	5	8.6 × 10^3^
7	R	78	Male	15.5	15	1.2 × 10^6^
8	R	77	Female	29.6	4	2.4 × 10^7^
9	L	59	Male	8.3	5	8.0 × 10^3^

We first evaluated whether cytotoxic T lymphocytes of corneal endotheliitis patients were capable of releasing interferon-γ or Granzyme B in response to CMV-infected corneal endothelial cells. As is consistent with the fact that endotheliitis patients are not immune compromised, CMV specific release of interferon-γ and Granzyme B were observed for cytotoxic T lymphocytes from endotheliitis patients (interferon-γ: 10 ± 19 folds, Granzyme B: 2.1 ± 2.3 folds).

### Efficacy of anti-viral CTL responses for corneal endothelial cells

We then examined whether the CMV-specific CTL cells may have anti-viral effect on the amount of DNA of CMV in the patients’ eye. For this, the maximum number of CMV DNA copies in the aqueous humor during relapses was determined for association with the CTL responses for CMV. We assessed the release of Granzyme B because CTL responses need to kill the infected cells directly as well as the virus.

A linear regression analysis indicated that the induction of CTL reduced the number of DNA copies of CMV significantly by almost one-half (log10 coefficient for CTL fold increase, − 0.25, *P* = 0.01) in endotheliitis patients. Thus, anti-viral CTL responses appeared to be operative in reducing the level of CMV.

### Alteration of CMV epitope dependent CTL responses in patients with corneal endotheliitis

**Figure 2:**
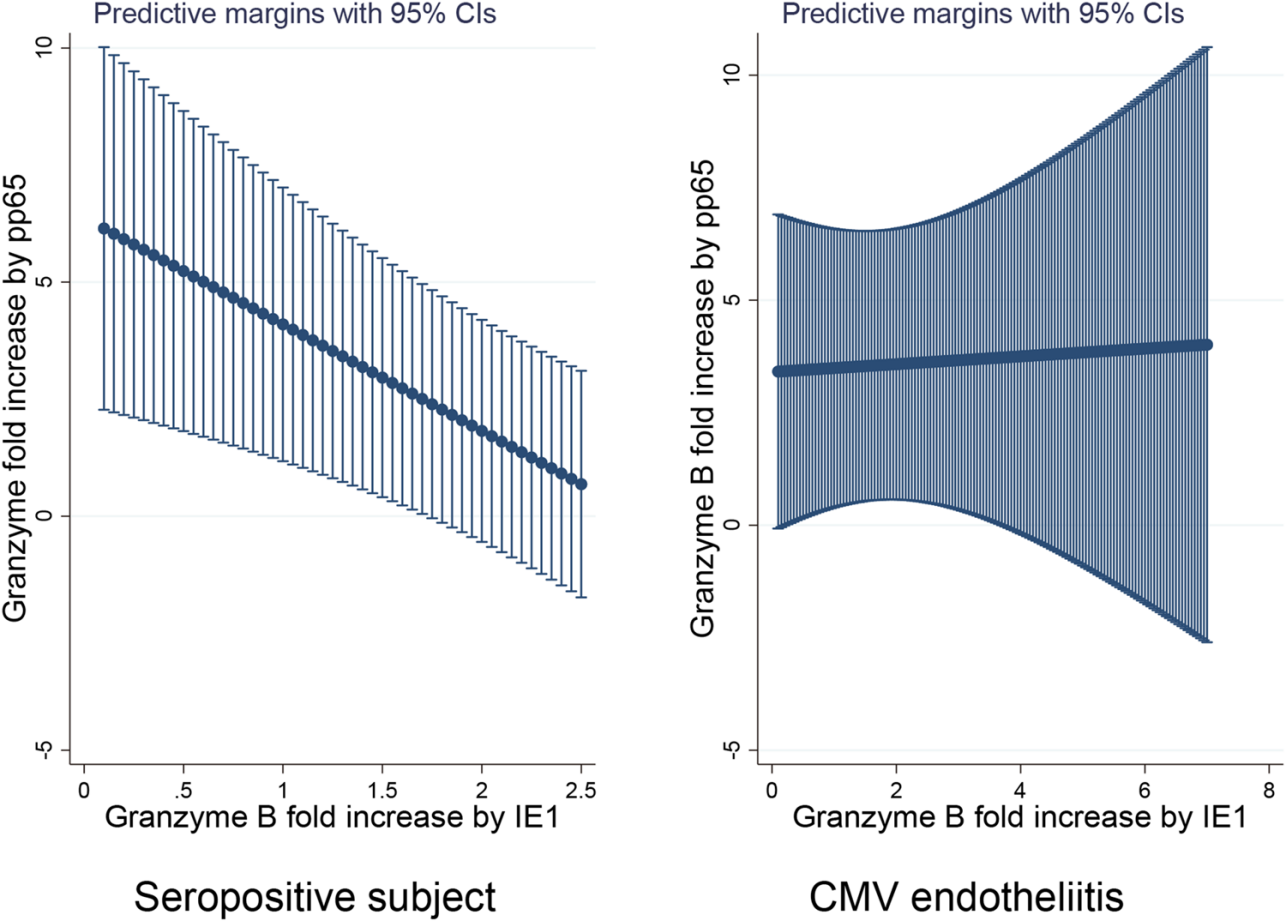
Reactivity profile of cytotoxic T lymphocyte (CTL) to cytomegalovirus (CMV) antigens in CMV endotheliitis and seropositive control subjects. The CTL activity was measured by the rate of increase of granzyme B against media as fold increases. The relationship of the induction of IE1 and pp65 specific CTL responses are calculated using linear regression analysis for CMV endotheliitis (*N* = 9) and seropositive before onset subjects (*N* = 6) after age adjustment. Seropositive subject showed higher pp65 CTL and low IE1 response (*P* = 0.006, linear regression analysis). Endotheliitis patients were not changed for pp65 CTL response by the effect of IE1 CTL (*P* = 0.01).

To determine the basis of the findings, we hypothesized that an alteration of the CMV epitope dependent CTL responses may have caused this disease. We evaluated representative CMV antigens, IE1 and pp65, for CTL. The mean fold induction of IE1- and pp65- specific CTL responses were 2.2 ± 2.2 fold and 2.1 ± 2.2 fold, respectively, for corneal endotheliitis patients. When the CTL reactivity profile for corneal endotheliitis patients was assessed in comparison with seropositive healthy subject using linear regression analysis after age adjustments, the seropositive subjects had higher pp65 CTL and lower IE1 response (*P* = 0.006, linear regression analysis after age adjustments, Fig. 2). In contrast, the pp65 CTL response in corneal endotheliitis patients was not changed for various levels of IE1 CTL (*P* = 0.01, linear regression analysis after age adjustment).

The calculated profile suggested an association of IE1 CTL and corneal endotheliitis, and thus we assessed the positivity of IE1-specific CTL responses for corneal endotheliitis using Bayesian logistic regression analysis. The estimated probability of positive association of corneal endotheliitis with IE1-specific CTL responses was 87.0% (after age adjustments).

### CMV epitope dependent effect of CTL responses for corneal endothelial cell loss

**Figure 3:**
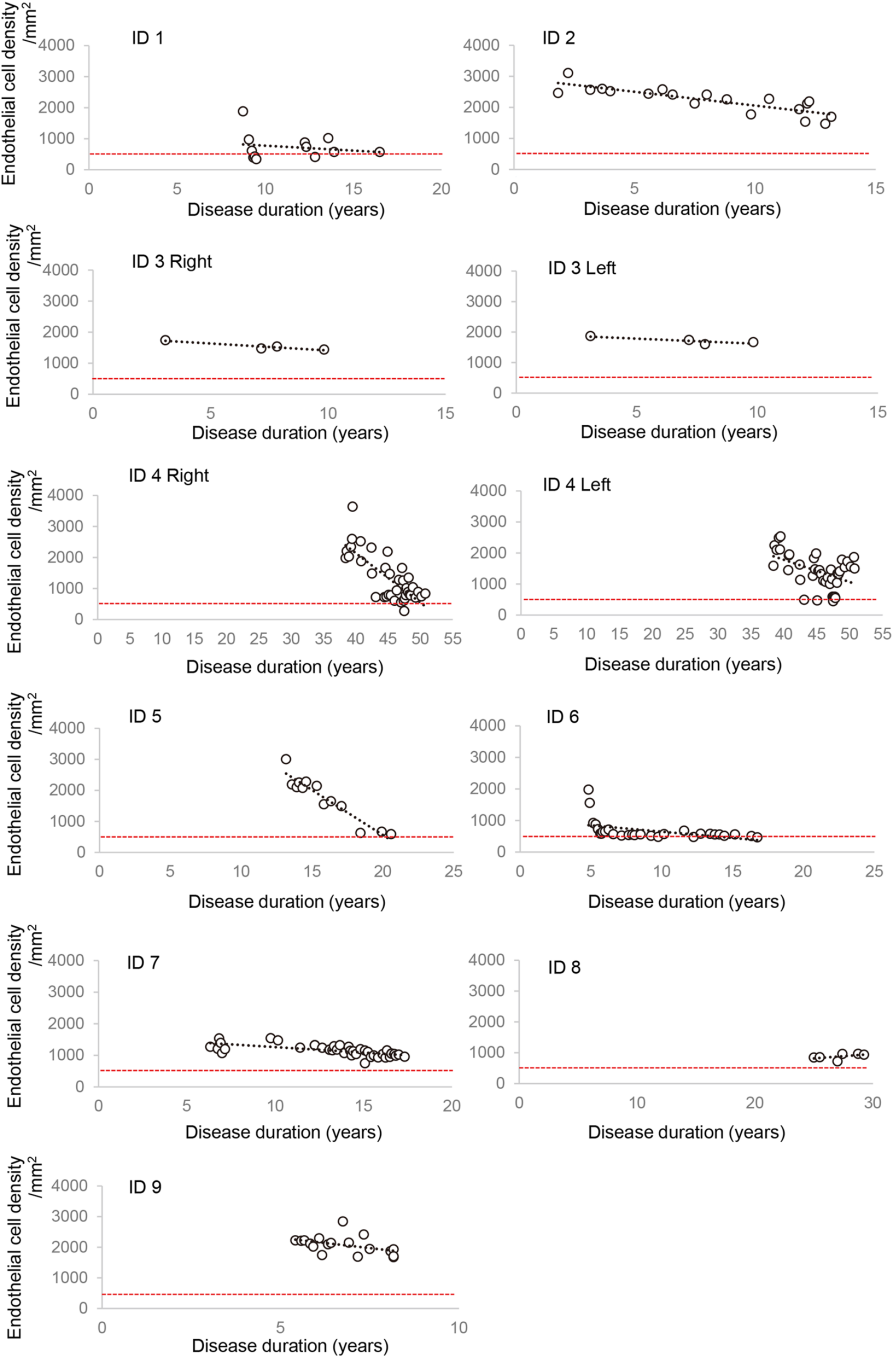
Corneal endothelial cell loss during the follow-up period in CMV endotheliitis patients. The corneal endothelial cell density was reduced during the long follow-up period. Red dotted line indicates endothelial cell density which represents when corneal transplantation would be necessary. All 9 cases are shown.

Long term viral infections of corneal endothelial cells can cause significant endothelial cell loss (Fig. 3). Thus, when the disease duration becomes longer, corneal transplantation will be necessary due to endothelial decompensation. Therefore, we analyzed the decrease of the endothelial cell density using a mixed linear regression analysis. The density of corneal endothelial cell was calculated to decrease by 79.73/mm^2^/year after age adjustments (*P* = 0.000, Table 3). This indicated that CMV endothelial patients will require corneal transplantation approximately 30 years after the disease onset.

**Table 3:** Association of virus-specific CTL and corneal endothelial cell loss by mixed linear regression analysis.

	Coefficient	*P* value	95% Confidence interval
Disease duration/year	− 79.73	0.000	− 8.09 ~ − 5.18
pp65-specific CTL/fold increase	447.94	0.000	344.99 ~ 550.88
IE1-specific CTL/fold increase	− 133.98	0.000	− 168.84 ~ − 99.14

We then examined how CTL or other clinical characteristics can affect the endothelial cells loss (Table 3). The induction of CTL for pp65 significantly improved the cell density by 448/mm^2^ per fold increase of CTL (*P* = 0.000).

Interestingly, the induction of IE1-specific CTL decreased the cell density by -133.98/mm^2^/fold increase (*P* = 0.000). This indicated that the induction of IE1-specific CTL damaged the endothelial cells and increased the need for corneal transplantation by 1.7 years /fold increase.

### Differential roles of CTL responses to prevent recurrences and surgical interventions

We next assessed how CTL responses affected the interval from onset to corneal transplantation using the Cox proportional hazard model (Table 4). Consistent with the earlier observations, the induction IE1-specific CTL significantly shortened the time to corneal transplantation with a hazard ratio/fold increase of 1.64 (*P* = 0.003). In contrast, the induction of pp65-specific CTL appeared to prolong the time to corneal transplantation with a hazard ratio/fold increase of 0.54 (*P* = 0.05).

**Table 4:** Association of virus-specific CTL and prognosis using Cox proportional hazard model.

	Hazard ratio	*P* value	95% Confidence interval
**Surgical intervention by corneal clarity loss**			
pp65-specific CTL/fold increase	0.54	0.05	0.30 ~ 1.01
IE1-specific CTL/fold increase	1.64	0.003	1.18 ~ 2.29
**Recurrence free time**			
pp65-specific CTL/fold increase	0.93	0.01	0.87 ~ 0.98
IE1-specific CTL/fold increase	0.87	0.13	0.72 ~ 1.04
**Surgical intervention by glaucoma**			
pp65-specific CTL/fold increase	1.08	0.480	0.87 ~ 1.36
IE- specific CTL/fold increase	1.47	0.001	1.18 ~ 1.83

Frequent recurrences are another characteristic of CMV corneal endotheliitis and increasing the recurrence free time is an important issue. We examined the association of recurrence free time and CTL induction using Cox proportional hazard model. Our results showed that pp65-specific CTL is protective, and significantly prolonged the recurrence free time with a hazard ratio/fold increase of 0.93 (*P* = 0.01 after age adjustment; Table 4). In contrast, IE1-specific CTL did not affect the recurrence free time with a hazard ratio/fold increase of 0.87 (*P* = 0.13).

Another characteristic of CMV endotheliitis is an elevation of the intraocular pressure (IOP). This is considered to be mediated by damage of the trabecular meshwork cells which often requires glaucoma surgery. When the CTL responses were analyzed for time to glaucoma surgery, the induction of IE1-specific CTL was significantly associated with reduced time to surgery (hazard ratio/fold increase: 1.47, *P* = 0.001; Table 4). Thus, IE1 CTL induction contributed to poor long-term control of the IOP.

## Discussion

Infections by the members of the herpes virus family including CMV, herpes simplex virus, and varicella-zoster virus are known to play major roles in corneal endotheliitis and refractory secondary glaucoma^10,12^. Of these, CMV infection of the anterior segment is especially refractory to conventional therapies and often requires surgical interventions^12^. The earlier evidence focused on CMV itself. PCR evaluations of the aqueous humor for viral DNAs revealed repeated reactivation of CMV in the eye presumably caused corneal endothelial cell loss and IOP elevation. However, the concept of pathogenicity of CMV have not provided sufficient explanation as to why healthy immunocompetent individual succumb to this refractory infection with repeated relapsing episodes. In this study, we focused on the protective arm of host factors and analyzed how the host reacts to CMV infections. We have presented evidence that a dysregulated protection of the host immune system allowed repeated viral reactivation causing the characteristic pathological signs of the disease.

Currently, most elderly subjects in the world have been infected with CMV. After the infection, the CMV genome persists in the cells, including the hematopoietic progenitors, in a state of latency after a primary infection. To control the latent CMV infection, anti-viral T cell responses are essential. The immune mechanism to prevent CMV reactivation is mediated by CD8 T lymphocytes and natural killer cells^13^. Earlier findings support the idea that CMV-specific CD8 T cells provide crucial systemic protection by reducing the circulating level of CMV virions^14^. Earlier, it was not clear whether this arm also contributed to the protection of the anterior segment CMV infections of the eye. Our results have confirmed that the induction of CMV-specific CTL was indeed associated with a reduction in the number of DNA copies of CMV in the eye. Importantly, our detailed analyses showed that insufficient priming of virus specific CTL allowed unrestricted virus reactivation. Moreover, the CTL responses were shown to probably be involved in corneal clarity loss or secondary glaucoma as collateral damage.

Generally, CMV-specific CTL responses are needed for the prevention of CMV diseases because the total number of T cells specific to CMV is very high in CMV seropositive individuals^14,15^. In the peripheral blood, 7.6% of the total CD8 T cells are CMV-specific, and they make up 12.8% of the memory compartment^15^. For the CTLs, IE1, IE2, and pp65 are the highest frequency antigens making up 2 to 3% of the memory CD8 subsets in the peripheral blood^15^, and pp65 and IE1 were well analyzed.

The protective effects of CTL have been well documented for stem cell transplantation (SCT) patients where the inductions of pp65 and IE1-specific CD8 CTL were shown to be associated with the absence of relapses of CMV DNAemia^16^. In addition, a recovery of these CTLs was associated with a lack of antigenemia in SCT patients^16^. In CMV-infected mice, CD8 T lymphocytes have been shown to also be important in preventing a reactivation of CMV because a depletion of CD8 T cells negated the protection from a reactivation of latent CMV^13^. Collectively, the proper development of virus-specific CTL can reduce the viral burden^14^.

In contrast to the results of earlier studies, we showed that the induction of IE1-specific CD8 CTL was associated with poor prognosis including corneal endothelial loss or IOP elevation. Seemingly protective CTL responses can be a pathogenic cause of diseases^17^. For this, the nature of the viral antigens and amount of infected cells appear to be the important determinants. This has been exemplified in the pathogenicity of CTL responses in hepatitis B virus infection leading to liver damage or cirrhosis^17^.

In CMV infections, IE1s are sporadically reactivated as an abundant immediate early protein without viral replication^18,19^. IE1 is stable and is initially transcribed after reactivation, and therefore easily recognized by the immune system^20–24^. Although IE1-specific T cell responses appear to reflect the extent of previous reactivations, the effectiveness of IE1 as a target epitope for vaccinations is known to be limited. In contrast, pp65 induces the most potent CMV-specific T cell response^9^.

CTL releases granzymes or perforins upon contact with the virus-infected cells. The released granzymes or FAS ligands on the CTLs destroy the infected cells or induce apoptosis of the infected cells for removal as well as chemokine induction, leading to inflammatory cell recruitment. Thus, this obvious imbalance in protective and damaging effect of targeting IE1 may explain why IE1 specific CTLs are associated with poor prognosis.

The pathogenic role of CTL has also been demonstrated for hepatitis B infections when the virus-specific CD8 T cells are essentially required for viral clearance^25^. The non-cytolytic arm reduces viremia whereas cytolytic CTLs destroy HBV-infected hepatocytes leading to liver pathogenesis and liver enzyme (sALT) release with inflammatory cell recruitment^26^.

For the development of vaccines for CMV disease, pp65 has been shown to be the most effective CTL epitope including patients with stem cell transplantation^9^. These and our findings suggest that pp65 may be a good candidate for a vaccination epitope to prevent corneal failure or recurrence of ocular CMV disease.

There are several limitations in interpreting the results. Our findings are based on a limited number of patients with specific HLA. The patients analyzed for CTL were chosen by HLA. This may cause inherent bias in interpreting the pathogenesis of this disease. For example, different reactivity to epitopes may be observed when a different HLA was chosen. However, unified and persistent analysis of CTL requires a stable cell line and not a primary culture of corneal endothelial cells. For this, we focused on HLA A*2402 reactivity for CTL because numbers of published reports are available for this reactivity for reference. In addition, we are aware that analysis on detailed epitopes profiles, not limited to IE1 and pp65, will be required for future development of vaccines.

However, CMV endotheliitis is a rare disease^6^, and no study has analyzed the roles of anti-viral CTL responses for this disease. We believe that our findings will advance the understanding of this visually devastating disease, and it will be the basis for more detailed analyses on epitope profile or designing longitudinal or cohort study in the future. This should then help develop efficacious management strategies for this refractory disease. In summary, altered profiles of antigen-specific CTL responses that are associated with corneal damage or secondary glaucoma are involved in the pathology of CMV endotheliitis.

## Methods

### CMV corneal endotheliitis patients

Diagnosis of anterior segment infection by CMV was made by detecting CMV DNA in the aqueous humor by real-time PCR and the clinical characteristics^6,12^.

An anterior segment infection by CMV includes anterior uveitis and corneal endotheliitis^6,12^.

The diagnosis of CMV corneal endotheliitis was made by detecting the DNA of CMV in aqueous humor samples^10^, and the following clinical findings; the presence of coin-shaped lesions, linear keratoprecipitates (KPs), and localized corneal edema with KP, chronic anterior uveitis, secondary glaucoma, and corneal endothelial cell loss^6^.

The corneal endothelial cell density of patients during the follow up visits was measured using noncontact specular microscopy (Noncon Robo FA-3809IID, Konan Medical Inc. Hyogo, Japan).

Immortalized human corneal endothelial cells (HLA A*2402) were used as antigen presenting cells to determine the corneal endothelial cell specific cytotoxic T lymphocyte (CTL) reaction^11^. To match MHC class I for CTL, endotheliitis patients were screened for HLA typing. Eleven eyes of 9 patients were typed as being positive for HLA-A*2402 and were enrolled between 2014 and 2018. All of the patients were examined at the Tottori University Hospital. For seropositive subjects as control, 6 healthy subjects with type A*2402 without eye diseases were studied for CTL profile. Their mean age was 51.0 ± 18.4 years. Seropositivity for CMV infection was confirmed by measurements of the level of serum antibody or the CTL reaction for CMV.

### Real-time PCR

The identification and quantification of the DNA of CMV in the aqueous humor were made by real-time PCR^10^. The DNA was extracted from the aqueous humor with the QIAamp DNA mini kit (Qiagen, Hilden, Germany), and the CMV glycoprotein B gene was amplified with the LightCycler (Roche, Basel, Switzerland) with the following primer sets.

Forward, AAGTACCCCTATCGCGTGTG;

Reverse, ATGATGCCCTCRTCCARGTC.

A standard curve was generated with known dilutions of the cloned DNA.

### Virus and corneal endothelial cells

The 20,040-4UAR strain of human CMV which can infect the corneal endothelial cells efficiently was used^11^. The CMV 20,040-4UAR strain was propagated on human foreskin fibroblast cells and was stored in aliquots at  − 80 °C as described^27^. Viral titers were measured by using the 50% tissue culture infection dose (TCID50) method^28^.

The availability of primary cultures of corneal endothelial cells to determine the MHC restricted CTL responses is limited, and their characteristics often change after prolonged culturing undergoing endothelial mesenchymal transition. Therefore, human corneal endothelial (HCEn) cells from a HLA-A*2402 positive donor was established as an immortalized HCEn cell line (generous gift from Dr Satoru Yamagami)^11^. The HCEn cells were plated on 96-well plates and grown to confluence in Dulbecco's modified Eagle's medium (Gibco, Grand Island, NY) supplemented with 10% fetal bovine serum.

### Viral antigen-specific CTL assay for human corneal endothelial (HCEn) cells

Peripheral blood mononuclear cells were isolated from heparinized blood of HLA-A*2402-positive patients using Ficoll density gradient centrifugation. Then, the CD8 + T lymphocytes were isolated using a magnet beads based negative selection kit (IMag, BD Bioscience, San Jose, CA), and a specific CD8 + T lymphocyte line was established using CMV (20,040-4UAR)-infected HCEn cells (HLA-A*2402)^11^. To stimulate the T cells, HCEn cells infected with CMV at MOI 0.1 for 24 h were irradiated and cocultured with CD8 + T lymphocytes for 14 days in RPMI supplemented with 10% fetal bovine serum and recombinant IL-2 (10 ng/ml).

To measure the CTL response, HCEn cells (4 × 10^4^ cells/well) were plated in 96 well plates. The HCEn cells were infected with CMV or pulsed with epitopes of CMV peptides to determine the degree of CMV-specific CTL response. These cells were used as antigen presenting cells for the CTL responses. HLA-A*2402-restricted peptides, SSAKRKMDPD and QYDPVAALF were used for the CMV epitopes of IE1 and pp65 respectively^29,30^.

Peptide-pulsed HCEn cells were co-cultured with the CD8 T cell line at CD8 + T cells/HCEn cells ratio of 1/3. The supernatant collected after 6 h were measured for the released of granzyme B using a commercially available ELISA kit (Thermo Fisher, Waltham, MA). The fold increase of granzyme B release was calculated as the ratio to granzyme B secreted from the CTL response without specific antigen stimulation. To confirm the MHC class I dependency of the granzyme B release, anti-MHC class I antibody, W6/32 (Biolegend, San Diego, CA) or control antibody, were used^11^.

All of the procedures used conformed to the tenets of the Declaration of Helsinki, and they were approved by the Institutional Review Board of Tottori University, Tottori, Japan. An informed consent was obtained from all of the participants.

### Statistical analyses

Data are presented as the means ± standard deviation. Posterior probability of positive association with antigen-specific CTL responses were estimated using Bayesian logistic regression analysis using the Metropolis–Hastings algorithm. The effects of antigen-specific CTL response on the longitudinal corneal endothelial cell loss were analyzed by generalized mixed linear regression model to assess hierarchical structure of the data. Hazard ratios of antigen-specific CTL on recurrence free time or time to surgical interventions were analyzed using Cox proportional hazard model. The data from patients with bilateral infections were analyzed as nested within the subject. Series of events were analyzed as nested within the eye. Statistical analyses were performed with Stata 17 (StataCorp, College Station, Texas). A *P* < 0.05 was considered significant.

## Data Availability

The datasets analyzed during the current study are available from the corresponding author on reasonable request.
